# Foliar Fertilizer Application Alters the Effect of Girdling on the Nutrient Contents and Yield of *Camellia oleifera*

**DOI:** 10.3390/life13020591

**Published:** 2023-02-20

**Authors:** Shuangling Xie, Dongmei Li, Zhouying Liu, Yuman Wang, Zhihua Ren, Cheng Li, Qinhua Cheng, Juan Liu, Ling Zhang, Linping Zhang, Dongnan Hu

**Affiliations:** 1 Jiangxi Provincial Key Laboratory of Silviculture, College of Forestry, Jiangxi Agricultural University, Nanchang 330045, China; 2 Jiangxi Agricultural University Library, Nanchang 330045, China

**Keywords:** *Camellia oleifera*, girdling, foliar fertilizer, nutrient content, yield

## Abstract

Improving the economic benefits of *Camellia oleifera* is a major problem for *C. oleifera* growers, and girdling and foliar fertilizer have significant effects on improving the economic benefits of plants. This study explains the effects of girdling, girdling + foliar fertilizer on nutrient distribution, and the economic benefits of *C. oleifera* at different times. It also explains the N, P, and K contents of roots, leaves, fruits, and flower buds (sampled in March, May, August, and October 2021) and their economic benefits. The results showed girdling promoted the accumulation of N and K in leaves in March 2021 (before spring shoot emergence) but inhibited the accumulation of P, which led to the accumulation of P in roots and that of N in fruits in August 2021 (fruit expansion period). Foliar fertilizer application after girdling replenished the P content of leaves in March 2021, and P continued to accumulate in large quantities at the subsequent sampling time points. The N and P contents of the root system decreased in March. In October (fruit ripening stage), girdled shrubs showed higher contents of N and K in fruits and flower buds, and consequently lower relative contents of N and K in roots and leaves but higher content of P in leaves. Foliar fertilizer application slowed down the effects of girdling on nutrient accumulation in fruits and flower buds. Spraying foliar fertilizer decreased the N:P ratio in the flower buds and fruits of girdled plants. Thus, foliar fertilizer spray weakened the effects of girdling on the nutrient content and economic benefits of *C. oleifera*. In conclusion, girdling changed the nutrient accumulation pattern in various organs of *C. oleifera* at different stages, increased leaf N:K ratio before shoot emergence, reduced root K content at the fruit expansion stage and the N:K ratio of mature fruit, and promoted economic benefits.

## 1. Introduction

*Camellia oleifera* Abel (Theaceae) is an evergreen shrub widely planted in 18 provinces and regions of China, including Hunan, Jiangxi, and Guangxi (in order of planting area) [1,2]. Unlike other fruit trees, *C. oleifera* is characterized by the coexistence of fruit and flowers as well as vegetative growth and reproductive growth. This results in different periods of distribution patterns of *C. oleifera* being different from other fruit trees. Therefore, scientific management of the nutrient content of *C. oleifera* is an important measure for maintaining its yield at high levels [1,3,4]. In recent years, scholars have conducted extensive research on the fertilization of *C. oleifera* forests [5]; however, fertilization in forestland was found to be difficult and expensive. Tree girdling, as a means of nutrient content regulation, has been widely used in citrus [6], grape [7], apple [8], kiwi [9], and other fruit trees [10]. The objective of girdling is to sever the phloem and prevent the flow of carbohydrates to the underground plant parts, thus promoting reproductive organ growth, flowering, and fruit development and quality [11,12]. However, girdling should be carried out when the nutrient content of the tree is sufficient. Foliar fertilizer is applied to plant stems and leaves so that plants can absorb various nutrients through stems and leaves and improve their nutritional status [12]. Meanwhile, with the application of unmanned aerial vehicles (UAVs) for plant protection [13,14], the application of foliar fertilizer on *C. oleifera* trees is in the initial stages. Foliar fertilizer will become another important way of nutrient management in *C. oleifera* [15].

A large number of experiments on fruit trees show that girdling effectively reduces the N(Nitrogen), P(Phosphorus), and K(Potassium) contents of the leaves above the girdle [11,16]. Therefore, if the nutrients are not replenished immediately after the girdling, the tree can become weak and eventually die. Therefore, reasonable foliar fertilizer application can effectively improve the nutrient content of plant organs and promote the growth and development of plants [15]. Urea has a high N content and is often used as a common fertilizer for plant nitrogen supplementation [17]. Potassium dihydrogen phosphate contains P and K, and the plant utilization rate is high, which can promote the absorption of N and P by plants, and it has good water solubility, which is the first choice for foliar fertilizer [18]. Girdling can reduce flower and fruit drop. Furthermore, plants are also sprayed with gibberellin [18], naphthaleneacetic acid [19], or boron [20] to reduce flower and fruit drop. *C. oleifera* has been cultivated for a long time [21]; however, its management is extensive, and there has been no way to report the application of girdling. In a preliminary experiment, we showed that girdling could effectively increase the fruit yield of *C. oleifera*. However, the effect of girdling on nutrient distribution in *C. oleifera*, the need to supplement nutrients after the application of girdling, and the relationship between the nutrient characteristics of *C. oleifera* and yield remain unclear. In this study, 10-year-old *C. oleifera* trees were treated with girdling and foliar fertilizer, and the root, leaf, flower, and fruit samples were collected to determine and analyze the key phenological period of *C. oleifera*. Additionally, ripe *C. oleifera* fruits were picked to determine fruit quality. To explore the effects of girdling and foliar fertilizer application on the nutrient content of *C. oleifera* trees, the distribution regulation and stoichiometric ratio characteristics of these trees were analyzed in different periods. Moreover, to develop recommendations and obtain theoretical support for nutrient management in *C. oleifera*, the relationship between nutrient characteristics and yield was analyzed via path analysis.

## 2. Materials and Methods

### 2.1. Plant Material and Study Site

Ten-year-old clones of *C. oleifera*, with an average plant height of 2.70 m, ground diameter of 76.83 mm, east-west crown width of 2.55 m, north-south crown width of 2.62 m, and plant row spacing of 2.0 m × 3.0 m, were used in this study. The experiment was conducted in the Jiu long shan Township of Yushui District, Xinyu City, Jiangxi Province, China (27°40′ N, 114°49′ E). The study site has a subtropical monsoon climate, with an average annual temperature of 15 °C and abundant annual rainfall of 1680 mm (Resources come from Xinyu Meteorological Bureau). The woodland was planted in strips on a gentle slope, and the soil contained 25.12 mg kg^−1^ available nitrogen, 5.73 mg kg^−1^ available phosphorus, and 20.25 mg kg^−1^ available potassium. All *C. oleifera* clones were planted in the same period and grown using the same management practices.

### 2.2. Study Design

A single factor experimental design was adopted. Girdling was applied at the flowering stage, and foliar fertilizer was sprayed after girdling. The treatment subjected to neither girdling nor spray foliar fertilizer served as the check control (CK). Each treatment contained 15 plants (45 plants total). Additionally, the treatments were implemented on three adjacent strips in the middle of the hillside, with one treatment per strip.

### 2.3. Experimental Method

#### 2.3.1. Girdling Technique

The experiment began in November 2020 (at the first flowering stage). *C. oleifera* shrubs showing uniform growth were selected and girdled 270° with a girdling cutter, completely severing the phloem but without damaging the xylem. The girdle was 2 mm wide, and was located on first-order branches at 10–20 cm above the main stem [22]. Finally, the shrubs were labeled according to the treatment.

#### 2.3.2. Foliar Fertilizer Spray Technique

A sprayer was used to spray the surface of *C. oleifera* leaves with a foliar fertilizer composed of 0.2% urea, 0.2% potassium dihydrogen phosphate, 0.2% borax, 50 mg L^−1^ gibberellin, and 20 mg L^−1^ naphthalene acetic acid (The concentration selection is determined on the basis of comprehensive consideration of previous studies). A total of 7.5 L of foliar fertilizer was applied to 15 *C. oleifera*, with an average of 0.5 L per tree. The fertilizer was sprayed 1 week after girdling on a day with no rain [23].

#### 2.3.3. Sample Collection

Plant organs were sampled in 2021; roots and leaves were sampled on 10 March (before spring shoot emergence), 25 May (after spring shoot emergence), 5 August (fruit expansion stage), and 15 October (fruit maturity stage); fruits were sampled only on 25 May and 15 October, and flower buds were sampled only on 15 October, because flower buds and fruits were either too small or not present at the other time points. Sampling was performed in triplicate on each date, with each replicate containing samples collected from five plants. After collection, the samples were brought to the lab, washed, fixed, dried to a constant weight, pulverized, and stored for testing.

On 15 October 2021, the yield per plant of *C. oleifera* was determined (all fruits from a single *C. oleifera* plant were picked separately, weighed, and recorded). At the same time, before fruit picking, 24 fruits were randomly picked in the upper, middle, and lower layers in the four directions of southeast, southwest, and northwest, with different treatments (one for every five trees, a total of three parts). After collection, it was brought back to the laboratory to determine the fresh weight of single fruit and single fruit seed kernels, and then the seed kernels were put into the oven and baked to constant weight, crushed with a mortar, and stored to determine the oil content of the seed kernels.

#### 2.3.4. Determination of Nutrient Content and Fruit Economic Characteristics

Before the test treatment, three parts of 0–20 cm rhizosphere soil were collected according to the “S” sampling method, dried naturally, passed through a 2 mm sieve, and sealed and stored.

The available nitrogen content was determined using the alkaline hydrolysis method [24]. The available phosphorus content was determined using the molybdenum blue colorimetric method [21]. The available potassium content was measured using an atomic flame photometer [1].

Weigh 0.1 g samples (leaves, roots, fruits, buds) and put them in a boiling tube, add H_2_SO_4_-H_2_O_2_, place a small funnel at the mouth of the tube, put it on the cooking furnace at 420 °C to boil until transparent, cool and set the volume to a 100 mL volumetric flask, let stand for 5–7 min, transfer to a 15 mL centrifuge tube for storage, and determine the concentrations of N, P, and K respectively.

The N content of various plant organs was determined using the automatic discontinuous analyzer (Smartchem 200, AMS, Rome, Italy) [22]; P content was determined using the molybdenum blue colorimetric method (GENESYS 180, Shanghai, China) [21]; and K content was determined with a flame photometer (FP6400, Shanghai, China) [1].

Weigh 0.5 g of the sample (dried seed kernels), put it into a folded filter paper packet, extract it using petroleum ether Soxhlet for 8 h, stand at 75 °C for 0.5 h, weigh the weight, calculate the oil content of seed kernels [1], the oil content of fresh fruits, and the oil yield per plant. The calculation formula is as follows:

Oil content of fresh fruit (%) = Oil content of seed kernels × Single fruit fresh weight(1)

Oil production per plant (g plant^−1^) = Oil content of fresh fruit × Yield per plant (2)

### 2.4. Data Analysis

The data were analyzed using SPSS 19.0. One-way analysis of variance (ANOVA), followed by Duncan’s multiple range test (DMRT), were used to identify significant differences (*p* < 0.05) in various parameters among the different treatments. Path analysis was used to determine the relationship between oil yield per plant and nutrient characteristics. Graphs were prepared in ORIGIN 2021.

## 3. Results

### 3.1. Effects of Girdling and Foliar Fertilizer Application on the Nutrient Contents of Various C. oleifera Organs

**Figure 1:**
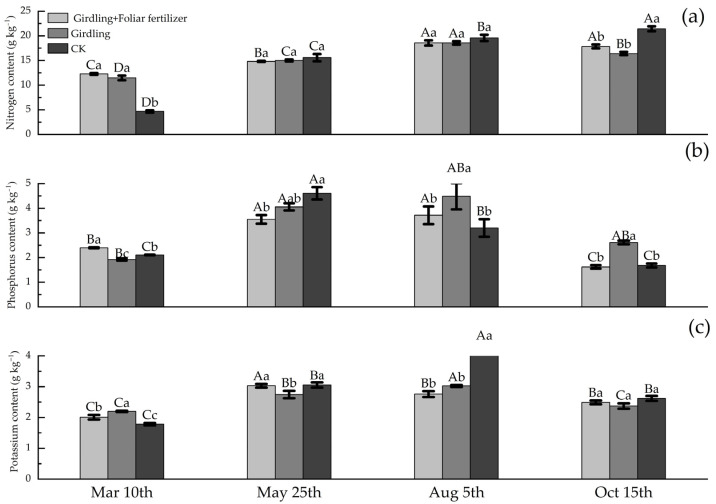
Nutrient content of *C. oleifera* leaves in different stages of each treatment. Data are means ± SE (n = 3), uppercase letters represent the difference between the same treatment and different periods (*p* < 0.05), lowercase letters represent the difference between different treatments in the same period (*p* < 0.05).

The N, P, and K contents of *C. oleifera* leaves in each treatment at four time points, 10 March (before spring shoot emergence), 25 May (after spring shoot emergence), 5 August (after fruit expansion), and 15 October (after fruit ripening), are shown in Figure 1. Without any treatment (CK), the N nutrient content of leaves was low before spring shoot emergence; however, the N nutrient content of new leaves gradually increased after spring shoot emergence, reaching a higher level at the later growth stage (Figure 1a). The girdling + foliar fertilizer treatments significantly increased the N content of leaves before spring shoot emergence. Compared with CK, the N content of girdling and girdling + foliar fertilizer treatments were 145.05% and 162.44% higher, respectively. During the period from spring shoot emergence to fruit expansion, no significant difference in leaf N content was detected between each treatment and CK. However, N did not accumulate in leaves at the fruit ripening stage; instead, it was transported to the fruit. At the fruit ripening stage, leaf N content in the girdling treatment was 12.66% lower than that in CK, which was significantly lower than that in the previous period. Spraying foliar fertilizer after girdling reduced the N output of mature leaves, and the gap in the N content between the girdling + foliar fertilizer treatment and CK was relatively smaller. The P content of the leaves in CK reached the highest after spring shoot emergence, and gradually decreased with fruit expansion, oil transformation, and flower bud differentiation at the later stage, indicating that P was transported from leaves to other organs (Figure 1b). Girdling decreased the P content by 8.82% compared with CK before spring shoot emergence. However, the P content of leaves increased during the period from the emergence of spring shoots to the expansion of fruits, and then decreased at the later stage. When the fruits matured, the P content of leaves was significantly higher than that in the CK by 55.54%. Spraying foliar fertilizer after girdling significantly increased the leaf P content before spring shoot emergence but inhibited the accumulation of P in leaves after spring shoot emergence. The leaf P content of the girdling + foliar fertilizer treatment was significantly lower than that of the girdling treatment at three stages after spring shoot emergence, but was not significantly different compared with CK.

The effect of the girdling and foliar fertilizer spray on K accumulation was different from that on the N and P accumulations. The K content of leaves in the CK treatment showed an increasing trend until the end of fruit expansion and decreased at fruit maturity (Figure 1c). Girdling promoted the accumulation of K in leaves before spring shoot emergence. However, during the period from spring shoot emergence to fruit expansion, the leaf K content in the girdling treatment was significantly lower than that in CK, and the K output also decreased at the later stage. At the fruit ripening stage, the K content of leaves in the girdling treatment was similar to that in the CK. Except during fruit expansion, foliar fertilizer spray after girdling reduced the difference in the leaf K content between the girdling and CK treatments at all time points. In other words, foliar fertilizer application weakened the regulation of girdling on the K content of *C. oleifera* leaves.

**Figure 2:**
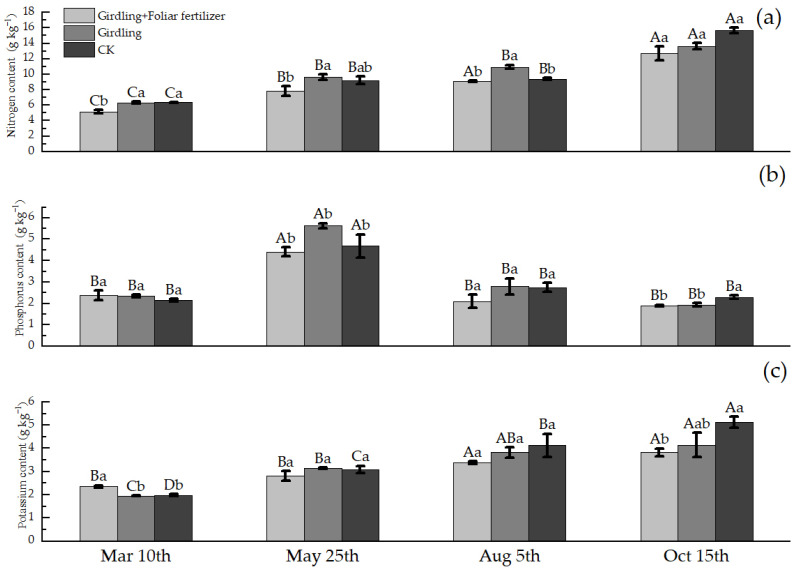
Nutrient content of *C. oleifera* roots in different stages of each treatment. Data are means ± SE (n = 3), uppercase letters represent the difference between the same treatment and different periods (*p* < 0.05), lowercase letters represent the difference between different treatments in the same period (*p* < 0.05).

With the change in root growth time, the N, P, and K contents of roots showed different dynamic regulation (Figure 2). Overall, the root N content increased after spring shoot emergence, slightly decreased during fruit expansion, and then increased at fruit maturity; the P content of roots increased at the emergence stage of spring shoot, and then decreased; and the root K content gradually increased with time. However, the change regulation of the different treatments was not completely consistent.

Girdling had no obvious effect on the root N content before and after spring shoot emergence (Figure 2a); however, the root N content was 16.44% after fruit expansion and 15.07% lower after fruit ripening compared with the CK. Therefore, girdling promoted N accumulation in the root system during fruit expansion but decreased N accumulation at fruit maturity. Application of foliar fertilizer after girdling reduced the root N content in each period, indicating that foliar fertilizer supplementation was not conducive to accumulation of N in the roots of girdled *C. oleifera* plants.

Girdling promoted the accumulation and output of root P at spring shoot emergence and fruit maturity (Figure 2b), respectively. The increase in root P was the largest (139.48%) before and after spring shoot emergence, and the decrease in root P was the largest (30.48%) after fruit maturation. Spraying foliar fertilizer after girdling inhibited the accumulation of P in the root system after spring shoot emergence. The root P content was 46.09% lower in the girdling + foliar fertilizer treatment than in the girdling treatment, which was similar to the root P content in CK. However, during other time periods, foliar fertilizer application after girdling had no obvious effect on the root P content.

Girdling had no significant effect of on the root K content in each period (Figure 2c). However, the application of foliar fertilizer after girdling significantly increased the root K content of spring shoots by 20.72% before emergence. Moreover, after the girdling treatment and until fruit ripening, the root K content was reduced by 17.87% and 25.32% after fruit expansion and ripening, respectively, compared with that of CK. The results indicated that spraying foliar fertilizer after girdling was not conducive to the accumulation of K in *C. oleifera* roots after spring shoot emergence.

**Figure 3:**
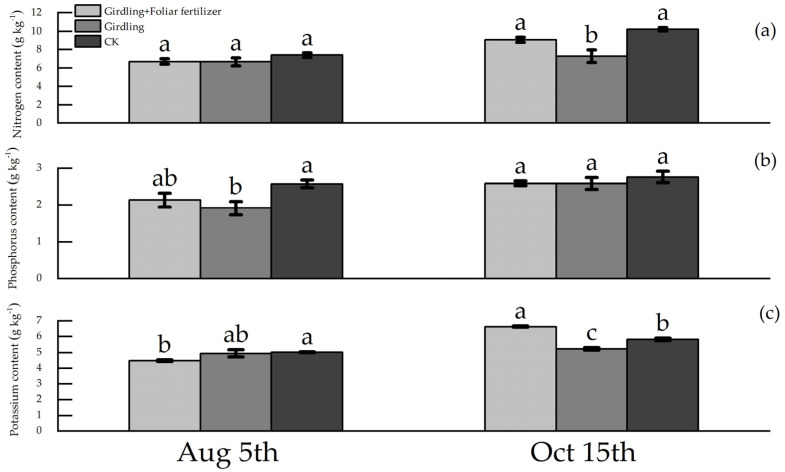
Nutrient contents of *C. oleifera* fruits in each treatment. Data are means ± SE (n = 3), lowercase letters represent the difference between different treatments in the same period (*p* < 0.05).

Girdling had no obvious effect on N and K accumulation in fruits at the fruit expansion stage (Figure 3). However, after fruit ripening, girdling reduced the N and K contents by 28.96% and 10.21%, respectively, compared with CK. The effect of girdling on the fruit P content was observed at the stage of fruit expansion, but little effect was noticed during fruit ripening. Application of foliar fertilizer after girdling reduced the N, P, and K contents of the fruit, and the N, P, and K contents of fruit at the swelling and ripening stages in the girdling + foliar fertilizer treatment showed no significant difference compared with the CK.

Girdling had no obvious effect on the N, P, and K contents of flower buds (Table 1). The N content of flower buds was increased in the girdling + foliar fertilizer treatment, which was 18.36% and 25.21% higher than that in the girdling and CK treatments, respectively. The P and K contents of flower buds were also increased in the girdling + foliar fertilizer treatment, but this increase was not significant compared with the girdling and CK treatments.

### 3.2. Effects of Girdling and Foliar Fertilizer on Nutrient Distribution in Different C. oleifera Organs

**Figure 4:**
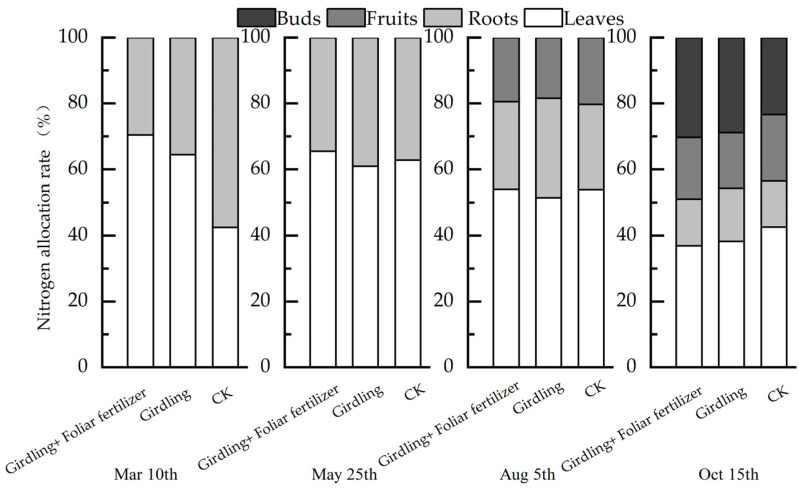
Nitrogen distribution characteristics of *C. oleifera* under different treatments in different periods.

Girdling and foliar fertilizer application changed the distribution pattern of N in *C. oleifera* organs (Figure 4). Before spring shoot emergence (10 March 2021), *C. oleifera* fruit had not developed, and flower buds were absent. In the CK treatment, the N nutrient content of roots was significantly higher than that of leaves, and the relative root N content was approximately 60%. Girdling caused more N nutrient accumulation in leaves. The relative N content of leaves in the girdling and girdling + foliar fertilizer treatments was more than 60%, and the relative N content of roots was less than 40%. However, the effect of girdling + foliar fertilizer was more obvious than that of the girdling treatment about the relative leaf N content. After the spring shoot emergence (25 May 2021), the fruit was small, and there were no flower buds. Girdling had little effect on N distribution in roots and leaves at this stage. After fruit expansion (5 August 2021), the root N content was the highest in the girdling treatment among all three treatments, and the relative N content of leaves, roots, and fruits in the girdling + foliar fertilizer treatment were consistent with those in CK. After fruit ripening (15 October 2021), the flower buds were swollen and about to bloom, and the relative N content of flower buds was lowest in CK and highest in the girdling + foliar fertilizer treatment. Additionally, on October 15, the relative N content of leaves was the highest in CK, indicating that girdling and foliar fertilizer application promoted the transport of N from leaves to flowers. Thus, girdling and foliar fertilizer application promoted the transport of N from leaves to other organs.

**Figure 5:**
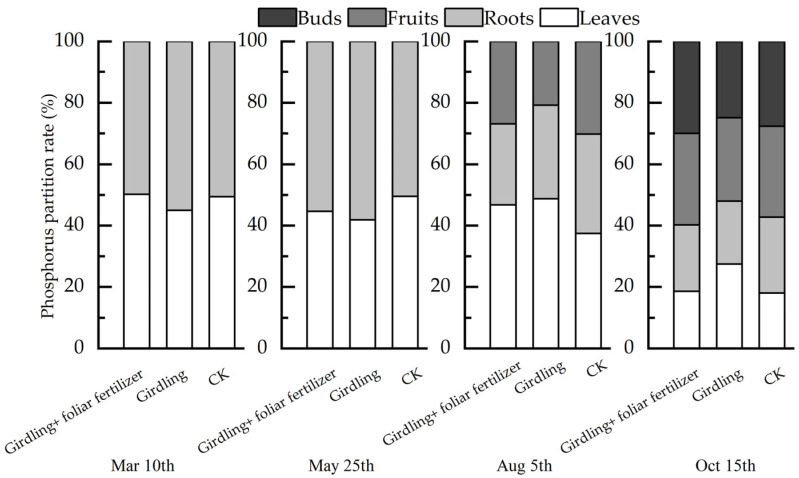
Phosphorus distribution characteristics of *C. oleifera* organs in different treatments in different periods.

The P distribution patterns in *C. oleifera* organs in different treatments at different stages were shown in Figure 5. At the spring shoot emergence stage, girdling decreased the content of P in leaves and increased P accumulation in the root system. The distribution of P in roots and leaves in the girdling + foliar fertilizer treatment was similar to that in CK. During the period after spring shoot emergence, the relative root P content was the highest in the girdling treatment, indicating that girdling was conducive to the accumulation of P in the root system. However, foliar fertilizer spray on girdled plants slightly decreased the relative root P content. After fruit expansion, the relative P content of fruits was the lowest, while that of leaves was the highest in the girdling treatment; however, the relative P content of roots showed no significant difference among the three treatments. At the fruit maturity stage, the relative P content of leaves was still the highest in the girdling treatment, and the P content in flower buds was relatively less, indicating that the girdling induced the transport of P from roots mainly to leaves. No significant difference was detected in P nutrient allocation between the girdling + foliar fertilizer and CK treatments. In general, the root system of *C. oleifera* accumulated more P in the first half of the year, and more P was transferred to the leaves in the second half of the year when fruits had expanded and ripened. This effect was alleviated by spraying foliar fertilizer after girdling.

**Figure 6:**
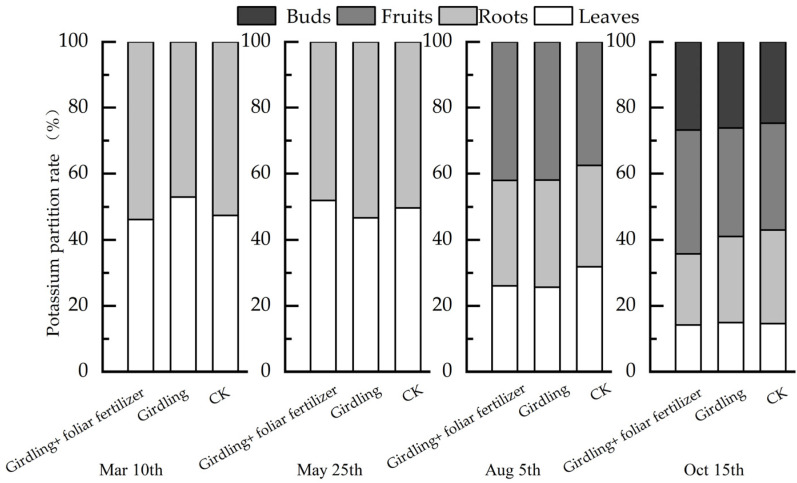
Potassium distribution characteristics of *C. oleifera* organs in different treatments in different periods.

Girdling and foliar fertilizer application also affected the distribution of K in various *C. oleifera* organs (Figure 6). Before spring shoot emergence, leaves showed a higher K content in the girdling treatment than in CK; however, spraying foliar fertilizer after girdling had little effect on the distribution nutrients in roots and leaves. After spring shoot emergence, girdling caused more K to accumulate in the root system, reducing the amount of K transported to the leaves. However, spraying foliar fertilizer after girdling further increased the K content of leaves. The relative K content of fruits in the girdling treatment increased with fruit growth and expansion. Foliar fertilizer application after girdling promoted the transport of K from roots and leaves to fruits at the fruit maturity stage, and the relative K content of fruit was highest in the girdling + foliar fertilizer treatment.

### 3.3. Effects of Girdling and Foliar Fertilizer Application on N:P and N:K Ratios in Different C. oleifera Organs

Girdling had no significant effect on the leaf N:P ratio from spring shoot emergence to fruit expansion, but significantly increased the leaf N:P ratio before spring shoot emergence and decreased the leaf N:P ratio after fruit ripening (Table 2).

The leaf N:P ratio in the girdling + foliar fertilizer was significantly lower than that in the girdling treatment but significantly higher than that in CK. During other periods, the effect of girdling + foliar fertilizer on the leaf N:P ratio was not obvious. In addition, during the leaf growth period, the N:P ratio in CK gradually increased, reaching a peak at the fruit maturity stage; however, in the other two treatments, the leaf N:P ratio first decreased and then increased at the fruit maturity stage. The root N:P ratio differed among the three treatments only in the first half of the year; after fruit expansion in the second half of the year, girdling had little effect on the root N:P ratio, regardless of the application of foliar fertilizer. The root N:P ratio decreased after girdling; however, after foliar fertilizer spray, the root N:P ratio decreased further, reaching levels significantly lower than those observed in CK, both before and after spring shoot emergence. The effect of girdling on the N:P ratio was not obvious at the fruit expansion stage, but was significant in mature fruits and flowers. Thus, in the CK treatment, the N:P ratio was significantly higher in fruits and significantly lower in flower buds. Application of foliar fertilizer after girdling increased the N:P ratio in mature fruits to different degrees, bringing the N:P ratio in fruits in the girdling + foliar fertilizer treatment closer to that in the CK treatment, although the N:P ratio in flower buds was higher in the girdling + foliar fertilizer treatment than in CK.

Compared with CK, the leaf N:K ratio was significantly higher in the girdling treatment before spring shoot emergence and fruit expansion, and lower after fruit ripening; however, the difference in the leaf N:K ratio was not a significant difference between these two treatments during late spring shoot emergence. Application of foliar fertilizer after girdling significantly increased the N:K ratio before spring shoot emergence but had little effect on the N:K ratio at the other stages. In *C. oleifera* roots, girdling had little effect on the N:K ratio before spring shoot emergence and after fruit ripening but significantly reduced the N:K ratio during spring shoot emergence and increased this ratio after fruit expansion. Application of foliar fertilizer after girdling significantly reduced the root N:K ratio before spring shoot emergence but had little effect on the N:K ratio at each stage after spring shoot emergence. Girdling had no obvious effect on the N:K ratio in fruits but increased the N:K ratio in flowers to varying degrees. Girdling + foliar fertilizer treatment showed a significantly higher N:K ratio in flower buds than CK.

### 3.4. Influence of Girdling and Foliar Fertilizer Application on C. oleifera Yield

Significant differences were observed in the fresh fruit yield, fruit oil content, and per-plant oil yield among the different treatments (Table 3). Girdling significantly increased the fruit yield, while spraying foliar fertilizer reduced the fruit yield increase; nonetheless, compared with CK, the girdling and girdling + foliar fertilizer treatments showed 63.56% and 33.24% higher fruit yield, respectively. However, girdling reduced the oil content of fresh fruit, and the application of foliar fertilizer after girdling further reduced the oil content; compared with CK, the girdling and girdling + foliar fertilizer treatments showed 13.07% and 20.03% lower oil yield, respectively. In addition, the per-plant oil yield in the girdling treatment was significantly increased by 41.91% and 33.65% compared with the girdling + foliar fertilizer and CK treatments, respectively.

### 3.5. Path Analysis of Nutrient Content, Nutrient Stoichiometric Ratio, and Per-Plant Oil Yield

Only the leaf N:K ratio in March, root K content in August, fruit N:P ratio in August, and fruit N:K ratio in October had significant effects on the per-plant oil yield, and the root P content in March and leaf N content in May had significant effects on per-plant oil production (Table 4). Among these effects, the effect of the N:K ratio on per-plant oil production per plant in March was positive, while those of the root K content in August, fruit N:P ratio in August, and fruit N:K ratio in October on the per-plant oil yield were negative.

The root K content in August, fruit N:P ratio in August, and fruit N:K ratio in October had direct negative effects on the per-plant oil yield (Table 5). However, the root K content in August, fruit N:P ratio in August, and fruit N:K ratio in October had indirect positive effects on the per-plant oil yield by interacting with each other and the leaf N:K ratio in March, which offsets the direct negative effects among factors. The leaf N:K ratio in March had an indirect negative effect on the per-plant oil yield by interacting with other factors. Among the effects of these factors, the direct positive effect of the leaf N:K ratio in March was greater, and the indirect positive effects of the fruit N:P ratio in August and fruit N:K ratio in October were greater. In addition, the residual path coefficient was 0.089, indicating that the factors in this equation fully explained the variation in oil production per *C. oleifera* plant.

## 4. Discussion

### 4.1. Effect of Girdling on the Nutrient Contents of C. oleifera Plants

Girdling is widely used in fruit trees [6,7,8,9] because it causes physical damage to phloem in the trunk, impeding the transport of carbohydrates and inorganic nutrients to the tree roots [25]. Studies show that girdling reduces the N, P, and K contents of leaves, and this effect is related to the healing time of girdling wounds [26]. The growth and development and the nutrient distribution pattern of *C. oleifera* are different from those of most fruit trees. The results of this study showed that girdling of the *C. oleifera* trunk in November significantly increased the N content of leaves before spring shoot emergence in early March of the next year, which was conducive to the accumulation of N in roots at the fruit expansion stage (August), and promoted the transport of N from leaves to fruits at the fruit maturity stage (October). During the period from the girdling of *C. oleifera* to the emergence of spring shoots, the fruits formed in the previous year (2020) had been harvested, and the new fruits (in 2021) had not yet formed; N had been transported to the leaves through the xylem; and competition from other organs was non-existent. Additionally, the girdle in the phloem had not yet begun to heal and could not transport N down to the roots, which significantly increased the N content of leaves during this time. After the girdle healed in mid-May, N was normally transported to above- and below-ground organs. After the girdling, the roots obtained more N and grew better, and the N output of the above-ground leaves increased at the later stage. In the first half of the year, P accumulated more in the roots, so that the P content of leaves before and after spring shoot emergence was significantly lower. However, after the fruit expansion and until the fruit maturity, P was transferred to the leaves in large quantities in the girdling treatment, resulting in a significantly higher leaf P content compared with CK. This result could be attributed to the P uptake characteristics of plants [21]. In *C. oleifera*, the first half of the year is the key period of P uptake and accumulation by roots. Before girdle healing, the normal transport of P is affected, resulting in a greater accumulation of P in the root system. After girdle healing, a large amount of P is transported to the leaves, greatly increasing the leaf P content.

In this study, girdling increased the availability of K to the leaves before spring shoot emergence, for the same reason as that which is responsible for N accumulation in leaves during this period. However, after spring shoot emergence, the K content of *C. oleifera* leaves, roots, fruits, and flowers in the girdling treatment was lower than that in CK, whereas the relative K content of flower buds and fruits was higher in the girdling treatment, so that more K was allocated to fruits and flower buds [27]. These results indicate that girdling promotes the distribution of K to fruits and flower buds, which is conducive to seed setting. However, it should be noted that girdling could consume a large amount of K while promoting seed setting, which may lead to insufficient K availability in the plant, thus requiring supplementation over time.

### 4.2. Influence of Ring Cutting on the Stoichiometric Ratios of N, P, and K in C. oleifera

N, P, and K are the limiting nutrients affecting plant growth, and the N:P and N:K ratios could be used as the determinants of plant health [28]. When the N:P ratio < 14, plant growth is limited by N; when the N:P ratio > 16, plant growth is limited by P; and when the N:P ratio = 14~16, plan growth is limited by both N and P [29]. The results of this study showed that the N:P of each organ of *C. oleifera* plants was less than 14 in different periods, indicating that the growth of *C. oleifera* was severely limited by N. In the trees girdled in November, the leaves had accumulated a large amount of N in March of the following year, whereas the level of P was low, improving the leaf N:P ratio, which to a certain extent, alleviated the effects of N limitation on *C. oleifera* growth. After the girdle wound healed, phloem returned to its normal transport capacity, gradually reducing the leaf N and P contents to levels consistent with those in the CK. The N:P ratio of the *C. oleifera* roots was relatively low before and after spring shoot emergence, which was severely restricted by N. At the later stage, the root N:P ratio gradually increased. At the fruit maturity stage, large amounts of N and P were accumulated in the vegetative organs before girdle wound healing, which were gradually transferred to the reproductive organs, flower buds, and fruits at the later stage. Therefore, the N:P ratio in *C. oleifera* fruits and flower buds was relatively high at the fruit maturity stage, which reduced the limitation of N in fruits and flower buds to a certain extent. When the N:K ratio is less than 2.1, plant growth is mainly limited by K [30]. The results of this experiment show that the N:K ratio in each organ of *C. oleifera* at different stages was less than 2.1, indicating that the growth of *C. oleifera* plants was limited by K. However, changes in the N:K ratio in different organs at different stages after girdling was the same as that in the N:P ratio, for roughly the same reasons.

### 4.3. Foliar Fertilizer-Induced Modification of the Effect of Girdling on the Nutrient Content of C. oleifera Organs

Application of foliar fertilizer after girdling affected nutrient accumulation in roots, increased the nutrient contents of fruits and flower buds, brought the N, P, and K contents of leaves, roots, and fruits closer to those in the CK, and weakened the regulatory effect of girdling on the contents of N, P, and K in *C. oleifera*. This result might be related to the vegetative growth of the above-ground parts of *C. oleifera* plants treated with foliar fertilizer [4]; the root system absorbed more nutrients. In addition, foliar fertilizer application after girdling also weakened the effects of girdling on the distribution of N, P, and K in various organs during the period from spring shoot emergence to fruit expansion and reduced the deviation of tree nutrient distribution patterns from the control in this period. Foliar fertilizer increased the relative contents of P and K in ripe fruits and flower buds of girdled *C. oleifera* trees, which may be related to the less fruits treated with foliar fertilizer [31].

### 4.4. Key Nutrient Characteristics of Girdled C. oleifera Trees for Increasing Yield

Girdling improved the fruit yield and per-plant oil yield of *C. oleifera*, but it did not increase the oil content of fresh fruit. It may be because girdling greatly increased the number of fruits per plant, resulting in reduced seed kernel oil content. However, the substantial increase in yield compensated for the reduction in seed oil content, leading to a substantial increase in oil production per plant.

Path analysis of *C. oleifera* nutrient contents and per-plant oil and fruit yield revealed that the root K content in August, fruit N:P ratio in August, and fruit N:K ratio in October were important factors affecting per-plant oil production. In March, the leaf N:K ratio had direct positive effects on oil production per plant, while the other three factors had indirect negative effects. This indicates that the increase in the leaf N:K ratio in March could directly and effectively promote the increase in oil yield per plant. Girdling promoted the accumulation of N in *C. oleifera* leaves in March, increasing the leaf N:K ratio. At the same time, girdling promoted a reduction in the K content of roots in August, and large amounts of N and P were accumulated in leaves, which were later transported to fruits, but the N and P contents of fruits were relatively low. In October, large amounts of N and K were accumulated in leaves and roots, which were later transported to fruits, and the amount of K transported was greater than that of N, resulting in low N and P contents of fruits.

## 5. Conclusions

Girdling and foliar fertilizer application affected the nutrient contents of *C. oleifera* organs at the flowering stage. The improvement in the economic benefits of girdling on *C. oleifera* is more obvious. In the girdling treatment, N was accumulated in leaves before girdle wound healing. However, the accumulation of N and K in the roots was limited. With the gradual healing of the girdle wound and the recovery of phloem transport capacity, the underground plant parts began to accumulate N. P accumulated in the root system before girdle wound healing. After wound healing, P began to accumulate in the leaves. At the fruit ripening stage, three nutrients (N, P, and K) previously accumulated in the vegetative organs were gradually transported to the reproductive organs (fruits and flower buds), and the amount of K transported was greater than that of N and P, which promoted flowering and fruiting. Compared with the girdling, the application of foliar fertilizer after girdling weakened the overall effect of girdling on nutrient regulation in *C. oleifera* trees but promoted the accumulation of a large amount of nutrients in roots, flower buds, and fruits.

## Figures and Tables

**Table 1:** Nutrient contents in flower buds of *C. oleifera* in each treatment.

Treatment	Nutrient		
	N Content (g kg^−1^)	P Content (g kg^−1^)	K Content (g kg^−1^)
Girdling+ foliar fertilizer	14.70 ± 0.73a	2.62 ± 0.14a	4.73 ± 0.15a
Girdling	12.42 ± 0.39b	2.35 ± 0.03a	4.15 ± 0.2a
CK	11.74 ± 0.21b	2.58 ± 0.17a	4.44 ± 0.12a
*p* value	0.013 *	0.353	0.145

Note Lowercase letters represent the difference between different treatments in the same period (*p* < 0.05); * indicates *p* < 0.05.

**Table 2:** The ratio of N:P and N:K of different organs in each period.

Date	Treatment	N:P				N:K			
		Leaves	Roots	Fruits	Buds	Leaves	Roots	Fruits	Buds
10 Mar	Girdling+ foliar fertilizer	5.12 ± 0.07b	2.19 ± 0.13b	–	–	6.11 ± 0.12a	2.19 ± 0.09b	–	–
	Girdling	5.98 ± 0.28a	2.69 ± 0.10a	–	−	5.21 ± 0.26b	3.24 ± 0.07a	−	−
	CK	2.22 ± 0.11c	2.96 ± 0.06a	−	−	2.63 ± 0.16c	3.20 ± 0.06a	−	−
25 May	Girdling+ foliar fertilizer	4.19 ± 0.17a	1.52 ± 0.06b	−	−	4.89 ± 0.13a	5.76 ± 0.07ab	−	−
	Girdling	3.71 ± 0.16a	1.28 ± 0.03c	−	−	5.49 ± 0.24a	5.14 ± 0.35b	−	−
	CK	3.41 ± 0.31a	1.79 ± 0.07a	−	−	5.11 ± 0.24a	5.85 ± 0.17a	−	−
5 Aug.	Girdling+ foliar fertilizer	5.11 ± 0.59a	5.75 ± 0.76a	3.19 ± 0.35a	−	6.74 ± 0.30a	5.84 ± 0.36ab	1.50 ± 0.06a	−
	Girdling	4.60 ± 0.91a	6.45 ± 0.96a	3.52 ± 0.36a	−	6.14 ± 0.08a	7.29 ± 1.75a	1.35 ± 0.13a	−
	CK	6.13 ± 0.27a	4.37 ± 0.43a	2.88 ± 0.18a	−	4.59 ± 0.02b	3.74 ± 0.36b	1.47 ± 0.05a	−
15 Oct.	Girdling+ foliar fertilizer	11.06 ± 0.61a	5.78 ± 0.01a	3.50 ± 0.14ab	5.63 ± 0.20a	7.17 ± 0.27b	7.50 ± 0.38a	1.37 ± 0.04a	3.10 ± 0.09a
	Girdling	8.11 ± 2.30b	5.82 ± 0.40a	2.79 ± 0.14b	5.27 ± 0.13b	6.93 ± 0.13b	6.54 ± 0.28a	1.38 ± 0.11a	3.01 ± 0.13ab
	CK	12.79 ± 0.46a	6.11 ± 0.26a	3.71 ± 0.18a	4.59 ± 0.25b	8.18 ± 0.22a	7.36 ± 0.18a	1.75 ± 0.03a	2.65 ± 0.03b

Note: Data are means ± SE (n = 3). Lowercase letters represent the difference between different treatments in the same period (*p* < 0.05).

**Table 3:** Yield of *C. oleifera* in each treatment, oil content of fresh fruit, and oil production per plant.

Treatment	Yield per Plant (kg Plant^−1^)	Oil Content of Fresh Fruit (%)	Oil Production per Plant (g Plant^−1^)
Girdling+ foliar fertilizer	9.58 ± 1.97ab	4.71 ± 0.20b	451.38 ± 85.56b
Girdling	11.76 ± 1.61a	5.12 ± 0.20b	603.29 ± 82.59a
CK	7.19 ± 1.67b	5.89 ± 0.23a	425.13 ± 99.18b
*p* value	0.173	0.000	0.013

Note: Data are means ± SE (n = 3). Lowercase letters represent the difference between different treatments in the same period (*p* < 0.05).

**Table 4:** Multiple stepwise regression analysis of oil production per plant and tree nutrients.

Item	Major Affecting Factors	Regression Equation	R^2^
Oil production per plant (g·plant^−1^) Y_1_	Leaf of N:K ratio in March X_1_	Y1 = 2164.853 + 175.036X_1_ − 206.702X_2_ − 213.266X_3_ − 320.795X_4_	0.992
	Root of K content in August X_2_		
	Fruit of N:P ratio in August X_3_		
	Fruit of N:K ratio in October X_4_		

**Table 5:** Path analysis of tree nutrient factors on oil production per plant of *C. oleifera*.

Independent Variable	Direct Path Coefficient	Indirect Path Coefficient					Residual Path Coefficient
		X1	X2	X3	X4	Total	
X_1_	1.065	--	−0.524	0.417	−0.959	−1.065	0.089
X_2_	−0.455	0.224	--	0.122	−0.281	0.065	
X_3_	−0.436	−0.171	0.117	--	0.262	0.208	
X_4_	−0.310	0.279	−0.191	0.186	--	0.274	

Note: X1 is N:K ratio of leaves in March, X2 is root of K content in August, X3 is N:P ratio of fruits in August, X4 is N:K ratio of fruits in October.

## Data Availability

The data that support the findings of this study are openly available in PubMed or available in other sources.

## References

[1] You L., Yu S., Liu H., Wang C., Zhou Z., Zhang L., Hu D. (2019). Effects of Biogas Slurry Fertilization on Fruit Economic Traits and Soil Nutrients of Camellia Oleifera Abel. PLoS ONE.

[2] He Z., Liu C., Zhang Z., Wang R., Chen Y. (2022). Integration of Mrna and Mirna Analysis Reveals the Differentially Regulatory Network in Two Different Camellia Oleifera Cultivars under Drought Stress. Front. Plant Sci..

[3] Ye H.-L., Chen Z.-G., Jia T.-T., Su Q.-W., Su S.-C. (2021). Response of Different Organic Mulch Treatments on Yield and Quality of Camellia Oleifera. Agric. Water Manag..

[4] Yu F., Xin M., Yao Y., Wang X., Liu K., Li Y. (2021). Manganese Accumulation and Plant Physiology Behaviour of *Camellia oleifera* in Response to Different Levels of Phosphate Fertilization. J. Soil Sci. Plant Nutr..

[5] He X. (2015). Effects of Foliar Fertilizers on Fruit Setting in Camellia Oleifera. Nonwood For. Res..

[6] Lu Z.-J., Yu H.-Z., Mi L.-F., Liu Y.-X., Huang Y.-L., Xie Y.-X., Li N.-Y., Zhong B.-L. (2019). The Effects of Inarching *Citrus reticulata* Blanco Var. Tangerine on the Tree Vigor, Nutrient Status and Fruit Quality of *Citrus sinensis* Osbeck ‘Newhall’ Trees That Have *Poncirus trifoliata* (L.) Raf. As Rootstocks. Sci. Hortic..

[7] Tyagi K., Maoz I., Lewinsohn E., Lerno L., Ebeler S.E., Lichter A. (2020). Girdling of Table Grapes at Fruit Set Can Divert the Phenylpropanoid Pathway Towards Accumulation of Proanthocyanidins and Change the Volatile Composition. Plant Sci..

[8] Baïram E., le Morvan C., Delaire M., Buck-Sorlin G. (2019). Fruit and Leaf Response to Different Source-Sink Ratios in Apple, at the Scale of the Fruit-Bearing Branch. Front. Plant Sci..

[9] Figiel-Kroczyńska M., Ochmian I., Lachowicz S., Krupa-Małkiewicz M., Wróbel J., Gamrat R. (2021). Actinidia (Mini Kiwi) Fruit Quality in Relation to Summer Cutting. Agronomy.

[10] Christopoulos M.V., Kafkaletou M., Karantzi A.D., Tsantili E. (2021). Girdling Effects on Fruit Maturity, Kernel Quality, and Nutritional Value of Walnut (*Juglans regia* L.) Alongside the Effects on Leaf Physiological Characteristics. Agronomy.

[11] Singh D., Dhillon W.S., Singh N.P., Gill P.P.S. (2015). Effect of Girdling on Leaf Nutrient Levels in Pear Cultivars Patharnakh and Punjab Beauty. Indian J. Hortic..

[12] de Lange J.H., Skarup O., Vincent A.P. (1974). The Influence of Cross-Pollination and Girdling on Fruit Set and Seed Content of Citrus ‘Ortanique’. Sci. Hortic..

[13] Mann M.S., Josan J.S., Chohan G.S., Vij V.K. (1985). Effect of Foliar Application of Micronutrients on Leaf Composition, Fruit Yield and Quality of Sweet Orange (*Citrus sinensis* Osbeck) Cv. Blood Red. Indian J. Hortic..

[14] Norozi M., ValizadehKaji B., Karimi R., Sedghi M. (2019). Effects of Foliar Application of Potassium and Zinc on Pistachio (*Pistacia vera* L.) Fruit Yield. J. Hortic. Sci..

[15] Duan W. (2015). Effect of Foliar Fertilizer on Leaf Anatomy Structure and Photosynthetic Characteristics of *Camellia oleifera* Container Seedlings. J. Northwest A F Univ..

[16] Yilmaz B., Çimen B., Incesu M., Yeşiloğlu T., Yilmaz M.I. (2018). Influence of Girdling on the Seasonal Leaf Nutrition Status and Fruit Size of Robinson Mandarin (*Citrus reticulata* Blanco). Appl. Ecol. Environ. Res..

[17] Li Y., Zou N., Liang X., Zhou X., Guo S., Wang Y., Qin X., Tian Y., Lin J. (2022). Effects of Nitrogen Input on Soil Bacterial Community Structure and Soil Nitrogen Cycling in the Rhizosphere Soil of *Lycium barbarum* L.. Front. Microbiol..

[18] Meng D., Xu P., Dong Q., Wang S., Wang Z. (2017). Comparison of Foliar and Root Application of Potassium Dihydrogen Phosphate in Regulating Cadmium Translocation and Accumulation in Tall Fescue (*Festuca arundinacea*). Water Air Soil Pollut..

[19] Sánchez-Ramos M., Berman-Bahena S., Alvarez L., Sánchez-Carranza J.N., Bernabé-Antonio A., Román-Guerrero A., Marquina-Bahena S., Cruz-Sosa F. (2022). Effect of Plant Growth Regulators on Different Explants of *Artemisia ludoviciana* under Photoperiod and Darkness Conditions and Their Influence on Achillin Production. Processes.

[20] Thakur P., Kumar P., Shukla A.K., Butail N.P., Sharma M., Kumar P., Sharma U. (2023). Quantitative, Qualitative, and Energy Assessment of Boron Fertilization on Maize Production in North-West Himalayan Region. Int. J. Plant Prod..

[21] Zeng J., Liu J., Lian L., Xu A., Guo X., Zhang L., Zhang W., Hu D. (2022). Effects of Scion Variety on the Phosphorus Efficiency of Grafted *Camellia oleifera* Seedlings. Forests.

[22] Levin A.G., Lavee S. (2005). The Influence of Girdling on Flower Type, Number, Inflorescence Density, Fruit Set, and Yields in Three Different Olive Cultivars (Barnea, Picual, and Souri). Crop Pasture Sci..

[23] Ma J., Zhang M., Liu Z., Wang M., Sun Y., Zheng W., Lu H. (2019). Copper-Based-Zinc-Boron Foliar Fertilizer Improved Yield, Quality, Physiological Characteristics, and Microelement Concentration of Celery (*Apium graveolens* L.). Environ. Pollut. Bioavailab..

[24] Wu F., Li J., Chen Y., Zhang L., Zhang Y., Wang S., Shi X., Li L., Liang J. (2019). Effects of Phosphate Solubilizing Bacteria on the Growth, Photosynthesis, and Nutrient Uptake of Camellia Oleifera Abel. Forests.

[25] Berüter J., Feusi M.E.S. (1997). The Effect of Girdling on Carbohydrate Partitioning in the Growing Apple Fruit. J. Plant Physiol..

[26] Huang D., Liu S., Zhou X., Qian D., Wu C., Xu Y. (2012). The Effect of Spiral Girdling on the Fruit-Setting and Development of Young Litchi Trees. Acta Hortic..

[27] Baninasab B., Rahemi M., Shariatmadari H. (2007). Seasonal Changes in Mineral Content of Different Organs in the Alternate Bearing of Pistachio Trees. Commun. Soil Sci. Plant Anal..

[28] Zhong Z., Zhang X., Wang X., Dai Y., Chen Z.X., Han X., Yang G., Ren C., Wang X. (2020). C:N:P Stoichiometries Explain Soil Organic Carbon Accumulation During Afforestation. Nutr. Cycling Agroecosyst..

[29] Yan Z., Kim N., Guo Y., Han T.-S., Du E.Z., Han W., Fang J. (2013). Effects of Nitrogen and Phosphorus Fertilization on Leaf Carbon, Nitrogen and Phosphorus Stoichiometry of *Arabidopsis thaliana*. Chin. J. Plant Ecol..

[30] Szczepanek M., Siwik-Ziomek A. (2019). P and K Accumulation by Rapeseed as Affected by Biostimulant under Different Npk and S Fertilization Doses. Agronomy.

[31] González C.E.A., Hernández M.M., Fernández-Falcón M. (2008). Nutrient Distribution and Stem Length in Flowering Stems of Protea Plants. J. Plant Nutr..

